# On the Optimal Temporal Resolution for Information Representation in Neural Activity: A Theoretical Analysis

**DOI:** 10.64898/2026.05.19.726394

**Published:** 2026-05-21

**Authors:** H. Fareed Ahmed, Toktam Samiei, Erfan Nozari

**Affiliations:** 1 Department of Mechanical Engineering, University of California Riverside, Riverside, CA, USA; 2 Department of Electrical and Computer Engineering, University of California Riverside, Riverside, CA, USA; 3 Department of Bioengineering, University of California Riverside, Riverside, CA, USA; 4 Neuroscience Graduate Program, University of California Riverside, Riverside, CA, USA

**Keywords:** Mathematical modeling, Neural code, Multiscale modeling, Information representation, Temporal integration, Averaging, Signal and noise correlations, Mesoscale optimality

## Abstract

**Introduction:**

Although neural activity is organized across temporal and spatial scales, the principles that determine the accuracy and fidelity of neural information representation across scales remain unclear. In particular, while recent empirical results have reported mesoscopic optimality in neural decoding, no theoretical accounts exist that explain when and why such intermediate scales emerge as optimal. Here, we develop an analytical framework to study the optimal temporal scale of information representation and its dependence on the dynamic structure of signal and noise in neural data.

**Materials and Methods:**

We formulate a multiscale theoretical model in which neural population activity is represented by temporally encoded trial vectors at microscopic, mesoscopic, and macroscopic resolutions. Neural responses are modeled as class-dependent mean activations (signal) corrupted by temporally correlated noise, and decay rates of correlations in both signal and noise are varied parametrically. Representational quality at each scale is quantified using the sensitivity index (d-prime) for decoding condition from neural activity.

**Results:**

We derive closed-form expressions for the sensitivity index at each temporal scale. These expressions reveal the key roles of signal and noise correlations as the main determinants of condition decodability at all scales. Comparing expressions under various combinations of signal and noise correlations reveals two regimes. First, when signal and noise correlations are absent or persistent over time, the optimal resolution falls at one of two extremes: macroscale (resp. microscale) if signal correlations are stronger (resp. weaker) than noise correlations. In contrast, when both signal and noise correlations decay with temporal separation, temporal integration produces a nontrivial trade-off: moderate integration improves decodability by suppressing noise while preserving coherent signal, whereas excessive integration degrades signal and decodability. Therefore, only in the latter regime, mesoscopic representations emerge as optimal across a broad range of biologically plausible parameters.

**Discussion:**

This work provides a theoretical explanation for how the optimal temporal scale of neural information representation depends on the interplay between signal and noise correlations and their temporal decay. Broadly, the framework establishes temporal integration as a principled mechanism linking multiscale neural dynamics to information representation and offers testable predictions across recording modalities and neural systems.

## INTRODUCTION

Neural activity evolves across a wide range of spatial and temporal scales, from millisecond spiking in individual neurons to slowly varying dynamics measured over large populations (Buzsáki, 2006; Bressler and Menon, 2010; Breakspear, 2017). This multiscale organization is one of the defining complexities of the brain, and understanding how information is distributed, transformed, and preserved across these spatiotemporal scales remains a fundamental problem in neuroscience. A central challenge is to determine how these scales are related, how information is represented differently at each scale, and which scale most effectively captures behaviorally relevant neural information.

Perhaps the most fundamental operation linking across spatiotemporal scales is averaging (integration). At the microscopic level, individual neurons integrate inputs across thousands of synapses, effectively averaging presynaptic activity over space, while membrane and synaptic time constants average activity over time (Cash and Yuste, 1999; Kandel et al., 2013). At larger temporal scales, fast spiking activity gives rise to slower collective dynamics such as low-frequency oscillations, which reflect the integrated behavior of neural circuits over extended time windows (Buzsáki, 2006). At larger spatial scales, coordinated population activity emerges from the interactions of many neurons, producing mesoscopic and macroscopic signals measurable at the level of local populations, cortical areas, or whole-brain recordings (Bressler and Menon, 2010; Breakspear, 2017). In all cases, integration serves as a biological bridge across scales, transforming fine-grained neuronal events into coarser yet often more stable representations of neural activity. As a fundamental operation embedded in neural processing itself, understanding integration is therefore crucial for studying the brain and interpreting brain-related signals.

Averaging is also a central tool in neural data analysis. Most preprocessing methods—including firing-rate estimation, peri-stimulus time histograms (PSTHs), low-pass filtering, dimensionality reduction, and population summaries—rely on averaging to improve signal-to-noise ratio and/or make neural activity more interpretable (Poldrack et al., 2011; Luck, 2014; Widmann et al., 2015). By suppressing trial-to-trial variability and reducing dimensionality, averaging can reveal structure that is otherwise obscured in noisy measurements.

Nevertheless, averaging can also blur fine temporal structure, wash out meaningful nonlinearities, and erase distinctions that are essential to the neural code. More generally, averaging involves an inevitable loss of information, as formalized by the data processing inequality (Cover and Thomas, 2006). Recent work has further shown that averaging can exert a strong linearizing effect, transforming functionally relevant nonlinearities into what appears as noise in macroscopic measurements (Nozari et al., 2024), a finding that was later theoretically demonstrated in (Ahmed et al., 2025; Ahmed and Nozari, 2023). The effect of averaging on neural representations, therefore, is not determined only by the amount or ratio of noise that is present in the data, but by how signal and noise are structured across space and time.

Specifically, the effect of temporal averaging depends primarily on the strength, distribution, and decay rate of correlations, *in both signal and noise*, over time. This in turn suggests that the optimal temporal scale of neural representation should be governed by the correlation structure of the underlying activity. If signal remains coherent across nearby time bins while noise decorrelates rapidly, temporal integration should improve representational quality by averaging-out noise faster than it averages-out signal. In contrast, averaging can lead to a rapid loss of information if nearby bins carry dissimilar signal, thereby combining weakly related or incompatible information into a single representation. As we demonstrated in (Samiei et al., 2024) using large-scale NeuroPixels recording in mice, biological population spiking activity appears to lie in the middle, with task-related information best decoded from *mesoscopic* representations. As with empirical studies in general, however, the findings of this study were limited to the animal model, brain regions, task conditions, and data modality that were used therein.

In this study, we develop a general theoretical framework for identifying the optimal temporal scale of information representation in neural activity as a function of the underlying statistical structure of signal and noise across time. Using a sensitivity index that captures within-class consistency, between-class separability, and trial-to-trial variability, we derive closed-form expressions for representational discriminability across scales, and use the latter to compare the accuracy of neural representations at micro-, meso-, and macroscopic scales.

Our analysis shows that the biological consequences of averaging depend critically on how signal and noise correlations decay (or not) over time. If signal remains statistically persistent across time while noise correlations decay, temporal integration favors the extremes—either microscopic or macroscopic, depending on the relative decay of noise correlations.; In contrast, when both signal and noise correlations decay with temporal distance, averaging generates a nontrivial trade-off, where limited integration strengthens coherent signal and suppresses variability, whereas excessive integration mixes weakly related signal components and accumulates unnecessary noise. Under these conditions, mesoscopic representations can indeed emerge as the optimal regime, as observed empirically in Samiei et al. (2024). Thus, our theory identifies the precise correlation structure under which intermediate temporal scales should be expected to maximize neural information.

More broadly, this work provides a theoretical foundation for interpreting temporal averaging as a biologically meaningful operation rather than a purely technical preprocessing step. It reframes the problem of neural coding across scales in terms of how information transforms under integration, and shows that the benefits and costs of averaging can be quantified through the interplay of temporally structured signal and noise. In doing so, it offers an analytical explanation for why intermediate temporal resolutions may be privileged in neural systems, and provides a principled framework for linking multiscale neural dynamics to information representation.

## RESULTS

Neural activity can be represented at multiple temporal resolutions, ranging from fine-grained (microscale) to fully aggregated (macroscale). As noted, we hypothesize that the optimal temporal scale emerges from a trade-off between the effects of averaging on signal and noise, governed by the structure of signal and noise correlations across time. To formalize and test this hypothesis, we develop a multiscale theoretical model that enables direct comparison of information representation across temporal resolutions, as described next.

### Theoretical framework for identifying optimal temporal resolution of neural activity

#### Context.

We begin by developing a framework for quantifying the effect of averaging on neural representations in the context of population coding of an extrinsic variable. Assume we have recorded the activity of N neurons at full (microscopic) resolution over a fixed window of b time points during a task with two conditions c=1,2. Let the N-dimensional *random vectors* $n_{i}^{c},\;i=1,\;2,\ldots ,b,\;c=1,2$ denote the population activations given each condition c. These can represent, for instance, the binary spiking activity of a population of neurons recorded via high density probes and binned at 1ms resolution, as in our prior work (Samiei et al., 2024). The two task conditions c=1 and c=2 can represent, e.g., two distinct sensory stimuli presented to the subject, or two disjoint groups of such stimuli, and we measure the amount of information present in ${\left\{n_{i}^{c}\right\}}_{i=1}^{b}$ by the accuracy of an optimal decoder that seeks to decode (classify) the extrinsic task condition based on the intrinsic neural activity ${\left\{n_{i}^{c}\right\}}_{i=1}^{b}$.

In this context, we measure and compare the effect of averaging on neural information by comparing the decoding accuracy of three classifiers, as follows. At the microscopic extreme, the classifier receives (a realization of) all population activity vectors

(1a) $$n_{1}^{c},n_{2}^{c},\ldots ,n_{b}^{c}$$

in order to predict the task condition c. At the opposite macroscopic extreme, the classifier receives only the fully integrated activity vector

(1b) $$\sum_{i=1}^{b}n_{i}^{c},$$

to make the same prediction. Finally, at the intermediate mesoscopic scale we provide the decoder with the two, partially-integrated vectors

(1c) $$\sum_{i=1}^{b/2}n_{i}^{c},\sum_{i=b/2+1}^{b}n_{i}^{c}.$$

Clearly, while the two extremes are uniquely defined, Eq. (1c) serves as a representative intermediate scale and can be changed or extended to other intermediate scales using the same approach. At each scale, we will quantify the accuracy of the optimal (Bayes) classifier and determine the scale that achieves maximum accuracy as a function of the signal and noise correlations in the data.

#### Decoder.

A unique feature, and challenge, in comparing decoding accuracies across scales is the potentially confounding effects of dimensionality. Unlike most machine learning problems where feature dimensions are either fixed or variable independently of the choice of model (due to missing data, e.g.), here the dimension of features available for decoding varies significantly and systematically with scale. As discussed in detail in (Samiei et al., 2024), most modern machine learning classifiers are highly sensitive to input dimensionality, often preferring medium to small input dimensions for the same amount of information present. Therefore, to decouple differences in information content from differences in dimensionality, we first encode (lift) available information at all scales to the same hyperdimension via a lossless linear encoding. As shown in Samiei et al. (2024), this can be achieved, at the microscale, by encoding the raw neural information of Eq. (1a) into the *trial hypervector*

(2a) $$v_{mic}^{c}=\sum_{i=1}^{b}{Sn}_{i}^{c}⊙t_{i}\in R^{D}.$$

In this expression $S\in R^{D\times N}$ is a matrix of spatial hypervectors that maps (lifts) neural activity into a hyperdimensional space $R^{D},\;D\gg Nb$. In other words, each column of S is a D-dimensional spatial hypervector assigned to (and preserving the identity of the spikes of) the corresponding neuron, and the vector ${Sn}_{i}^{c}$ provides the superposition of all such hypervectors weighted by the activations of their corresponding neurons. Similarly, the vectors $t_{i}\in R^{D}$ denote temporal hypervectors that assign a distinct identity to each time point, and the element-wise product ⊙ 'binds' (associates) the spatially-encoded hypervector ${Sn}_{i}^{c}$ with the temporal hypervector $t_{i}$. The final summation 'bundles' the resulting hypervectors over time, forming a single trial hypervector that contains information from all ${\left\{n_{i}^{c}\right\}}_{i=1}^{b}$. As shown in (Samiei et al., 2024, Supp. Note 1), Eq. (2a) constitutes a lossless mapping such that all $\left\{n_{i}^{c}\right\}$ can be recovered from $v_{\text{mic}}^{c}$ if the encoding (spatial and temporal) hypervectors are properly chosen and D is sufficiently large (D→∞ for ideal reconstruction).

Throughout this work we assume that the encoding hypervectors are properly chosen (e.g., consisting of independent and identically distributed elements draw from a zero-mean distribution (Samiei et al., 2024)) and fixed, and can therefore be treated as deterministic. If so, as D→∞, such hypervectors become asymptotically orthogonal. This implies, in particular, that $S^{⊤}S\approx \gamma I_{N}$, where γ∈R is a constant (squared norm of each spatial hypervector).

Similarly, the raw information in Eq. (1b) and Eq. (1c) are encoded into same-dimensional trial hypervectors

(2b) $$v_{mac}^{c}=S\left(\sum_{i=1}^{b}n_{i}^{c}\right)⊙t,$$

(2c) $$v_{mes}^{c}=S\left(\sum_{i=1}^{b/2}n_{i}^{c}\right)⊙t_{1}+S\left(\sum_{i=b/2+1}^{b}n_{i}^{c}\right)⊙t_{2},$$

respectively. Similar to the hyperdimensional encodings at the microscale, t denotes the temporal hypervector associated with the full (integrated) trial at the macroscale, while $t_{1},\;t_{2}$ are near-orthogonal hypervectors associated with the two halves of the trial, respectively.

It is well-known that the encoding (lifting) of information into hyperdimension also simplifies classification and allows for pattern recognition via simple inner products (Kanerva, 2009; Rahimi and Recht, 2007; Cortes and Vapnik, 1995). As such, let s∈𝒮={mic,mes,mac} denote any of the three resolutions, c∈{1,2} denote either condition, and $v_{s}^{c}$ and ${\overset{ˆ}{v}}_{s}^{c}$ denote two independent and identically distributed (i.i.d.) trial hypervectors from the same distribution corresponding to condition c. Then, regardless of the exact decoding architecture used, decoding accuracy is fundamentally limited by the *within-class* and *between-class* similarities

(3a) $$P_{s}^{c}=v_{s}^{c⊤}{\overset{ˆ}{v}}_{s}^{c},\;c=1,\;2,s\in 𝒮$$

(3b) $$P_{s}^{x}=v_{s}^{c⊤}{\overset{ˆ}{v}}_{s}^{c^{\prime }},\;c^{\prime }\neq c,$$

respectively. Note that both $P_{s}^{c}$ and $P_{s}^{x}$ are random variables (inheriting their randomness from that of ${\left\{n_{i}\right\}}_{i=1}^{b}$), and the amount of separation between their distributions controls how accurately one can distinguish between the two conditions. If trial hypervectors are highly similar within each condition (large $P_{s}^{c}$) but not across conditions (small $P_{s}^{x}$), decoding accuracy will be high. If, on the other hand, trial hypervectors are equally similar to each other within and between conditions, decoding accuracy will be at chance.

Accordingly, we will measure the decodability of conditions at each scale via the sensitivity index

(4) $$d_{s}^{\prime }=\frac{E\left[P_{s}^{c}\right]\;-\;E\left[P_{s}^{x}\right]}{\sqrt{\frac{1}{2}(Var\left(P_{s}^{c}\right)\;+\;Var\left(P_{s}^{x}\right))}},$$

where E[⋅] and Var(⋅) denote mean and variance, respectively. We use d-prime instead of the decoding accuracy of any specific classifier given the former's direct relation to the accuracy of an ideal (Bayes' optimal) decoder Averbeck and Lee (2006). Thus, in what follows, we will derive mathematical expressions for $d_{s}^{\prime }$ at each scale s∈𝒮 and study how they are related to each other depending on the correlation structures present in the data.

### Statistical Model.

As noted in the Introduction, we hypothesize that the effect of averaging on neural information depends on the relative strength of temporal correlations in each of signal (condition-relevant information) and noise (condition-irrelevant variability). To formally separate the two, let

(5) $$n_{i}^{c}=\mu_{i}^{c}+\sigma y_{i}^{c},$$

where random vector $n_{i}^{c}$ denotes the raw neural activity vector at time i under condition $c,\;\mu_{i}^{c}=E\left[n_{i}^{c}\right],\;\sigma^{2}=Var\left(n_{i}^{c}\right)$ for all i=1,…,b, and $y_{i}^{c}$ is the remaining normalized (zero-mean and identity-covariance, but not necessarily Gaussian) variability in $n_{i}^{c}$. In what follows, we will refer to $\mu_{i}^{c}$ and $\sigma y_{i}^{c}$ as the *signal* and *noise* components of $n_{i}^{c}$, respectively.

Throughout this work we assume that noise correlations decay exponentially with temporal distance. In particular, let $Y^{c}={\left[{\left(y_{1}^{c}\right)}^{⊤},\ldots ,{\left(y_{b}^{c}\right)}^{⊤}\right]}^{⊤}\in R^{Nb}$ represent the concatenation of all normalized noise vectors across the trial. We assume

(6a) $$Cov\left(Y^{c}\right)=\Sigma_{t}⊗I_{N},\;c=1,\;2,$$

where ⊗ denotes the Kronecker product, $I_{N}$ is the N×N identity matrix, and $\Sigma_{t}$ is the b×b temporal covariance matrix

(6b) $$\Sigma_{t}=\left[\begin{array}{ccccc} 1 & \rho & \rho^{2} & \ldots & \rho^{b-1}\\ \rho & 1 & \rho & \ldots & \rho^{b-2}\\ \rho^{2} & \rho & 1 & \ldots & \rho^{b-3}\\ \vdots & \vdots & \vdots & \ddots & \vdots \\ \rho^{b-1} & \rho^{b-2} & \rho^{b-3} & \ldots & 1 \end{array}\right],\;0\leq \rho <1,$$

The parameter ρ denotes the decay rate of noise correlations over time, and constitutes one of the key parameters in determining the optimal resolution for information decoding. Throughout this work we will assume the noise to be independent across the two conditions, i.e., $Cov\left(Y^{1},Y^{2}\right)=0$.

Equally important in the analyses of optimal temporal resolutions is the decay rate of *signal correlations* across time. We quantify signal correlations across time using the cosine similarity $\frac{\left\langle \mu_{i}^{c},\mu_{j}^{c}\right\rangle }{\left\|\mu_{i}^{c}\|\|\mu_{j}^{c}\right\|}$ between the signal components $\mu_{i}^{c}$. In particular, we consider two main scenarios, namely, one in which signal correlations decay with distance similar to Eq. (6),

(7a) $$\frac{\left\langle \mu_{i}^{c},\;\mu_{j}^{c}\right\rangle }{\left\|\mu_{i}^{c}\|\|\mu_{j}^{c}\right\|}=\beta^{\left|j-i\right|},\;0\leq \beta <1,$$

and an opposite scenario where signal correlations persist throughout the trial duration,

(7b) $$\frac{\left\langle \mu_{i}^{c},\;\mu_{j}^{c}\right\rangle }{\left\|\mu_{i}^{c}\|\|\mu_{j}^{c}\right\|}=\beta \;\text{for}\;\text{all}\;i\neq j,\;0\leq \beta <1.$$

In both scenarios, the parameter β plays the dual role to ρ and, as we will see, governs the impact of averaging on signal content.

Together, this formulation enables a systematic analysis of how temporal aggregation influences discriminability across multiple scales, with the parameters ρ and β governing the temporal structure of noise and signal, respectively, and, in turn, the effect of averaging on neural information representations.

### Information Representation at the Microscale

We begin the mathematical analysis of neural information content and decodability across temporal scales by deriving a mathematical expression for sensitivity index (d-prime) in Eq. (4) at the finest scale. To enable a tractable analytical characterization of the inner-product statistics, we introduce the following assumption, which will be maintained throughout the subsequent analysis.

#### Assumption 1.

The signal and noise components of neural responses are uncorrelated, i.e.,

$$\mu_{i}^{c⊤}y_{i}^{c}=y_{i}^{c⊤}\mu_{i}^{c}=0,$$

for all time points i and conditions c. □

Assumption 1 formulates the intuition that the task-*relevant* and task-*irrelevant* components of neural responses vary independently across the population. In the results that follow, this assumption further simplifies analytical expressions by allowing inner-product statistics to decompose into signal and noise contributions. That said, similar results can still be derived without this assumption, as shown in Supplementary Note 2 for a simple case.

We are now ready to state our first main result, providing a simple analytical expression for the sensitivity index (d-prime) at the microscopic resolution. The full proof of the result is deferred to Supplementary Note 1, with a proof sketch provided here for the interested reader.

#### Theorem 1 (Sensitivity Index at the Microscale).

Let $d_{\text{mic}}^{\prime }$ denote the sensitivity index defined in Eq. (4) at the microscale, with similarity statistics $P_{\text{mic}}^{c}$ and $P_{\text{mic}}^{x}$ defined in Eq. (3), trial hypervectors $v_{\text{mic}}^{c}$ defined in Eq. (2a), and neural responses following the statistical model in Eq. (5). Assume that Assumption 1 holds. Then,

(8) $$d_{mic}^{\prime }=\frac{\sum_{i=1}^{b}\left({\left\|\mu_{i}^{c}\right\|}^{2}\;-\;\langle \mu_{i}^{c},\mu_{i}^{c^{\prime }}\rangle \right)}{\sqrt{\sigma^{4}N(b\;+\;2E)}},$$

where

(9) $$E=\frac{(b\;-\;1)\rho^{2}\;-\;b\rho^{4}\;+\;\rho^{2b+2}}{{\left(1\;-\;\rho^{2}\right)}^{2}}.$$

#### Proof sketch.

In the interest of brevity we only outline the main steps of the derivation here; for the full proof see Supplementary Note 1. The core idea is that, at the microscale, the orthogonality of the temporal basis vectors $t_{i}$ and the columns of the spatial hypervectors matrix S, allows the inner products between trial representations to decompose into a sum of independent contributions across temporal bins. Using the decomposition in Eq. (5),

(10) $$P_{mic}^{c}=\gamma \sum_{i=1}^{b}{\left(\mu_{i}^{c}+\sigma y_{i}^{c}\right)}^{⊤}\left(\mu_{i}^{c}+\sigma {\overset{ˆ}{y}}_{i}^{c}\right),$$

(11) $$P_{mic}^{x}=\gamma \sum_{i=1}^{b}{\left(\mu_{i}^{c}+\sigma y_{i}^{c}\right)}^{⊤}(\mu_{i}^{c^{\prime }}+\sigma {\overset{ˆ}{y}}_{i}^{c^{\prime }}).$$

Using the statistical properties of trial hypervectors and their components, the expectations of within-class and between-class similarities reduce to,

(12) $$E\left(P_{mic}^{c}\right)=\gamma \sum_{i=1}^{b}{\left\|\mu_{i}^{c}\right\|}^{2},\;E\left(P_{mic}^{x}\right)=\gamma \sum_{i=1}^{b}\left\langle \mu_{i}^{c},\mu_{i}^{c^{\prime }}\right\rangle .$$

The variance terms in Eq. (4) depend, expectedly, on the noise terms in Eq. (5). Using the structure of the covariance matrix in Eq. (6), i.e., $\Sigma_{t_{ij}}=\rho^{\left|j-i\right|}$, we obtain:

(13) $$Var\left(P_{mic}^{c}\right)=Var\left(P_{mic}^{x}\right)=\gamma^{2}\sigma^{4}N\left(b+2\sum_{1\leq i<j\leq b}\rho^{2(j-i)}\right).$$

Evaluating the finite sum in Eq. (13) yields the closed-form expression for E in Eq. (9), and substituting these into the definition of $d_{\text{mic}}^{\prime }$ in Eq. (4) leads to the closed-form expression in Eq. (8). □

It is instructive to analyze the structure of $d_{\text{mic}}^{\prime }$ in Eq. (8) and what it reveals about information representation at the microscale. At this scale, $d_{\text{mic}}^{\prime }$ incorporates independent contributions across time bins, wherein the numerator of Eq. (8) represents the total integrated signal evidence accumulated through temporal summation. This reflects a setting where information is encoded locally in time, without further interactions between different temporal components. In contrast, the denominator of Eq. (8) represents integrated noise variance, incorporating temporal correlations through the factor b+2E and thus indicating that variability is not purely independent, but rather spreads across time. As a result, microscale representations exhibit noise contributions that scale linearly with the number of time points as the orthogonal basis vectors $t_{i}$ prevent the cross-temporal coupling of trial-to-trial fluctuations, while signal remains strictly additive and localized.

Theorem 1 characterizes the decodability of information representations at the finest temporal resolution, where each bin contributes independently. In the following section, we extend this analysis to coarser temporal scales, deriving analogous expressions for mesoscale and macroscale representations obtained through temporal aggregation. We will subsequently compare these results and study the conditions under which each scale provides the most amount of task-relevant information.

### Information Representation at Integrated Scales

Building on the microscale analysis described above, we next consider the *mesoscale representation* obtained by aggregating temporal bins into two blocks of size b/2 time points each. The following result provides a closed-form expression for the sensitivity index $d_{\text{mes}}^{\prime }$, clarifying the precise contributions of signal and noise components to information decodability. The proof sketch is similar to that of Theorem 1, and the full proof is provided in Supplementary Note 1.

#### Theorem 2 (Sensitivity Index at the Mesoscale).

Let $d_{\text{mes}}^{\prime }$ denote the sensitivity index defined in Eq. (4) at the mesoscale, with similarity statistics $P_{mes}^{c}$ and $P_{mes}^{x}$ defined in Eq. (3), trial hypervectors $v_{mes}^{c}$ defined in Eq. (2c), and neural responses following the statistical model in Eq. (5). Assume that Assumption 1 holds. Then,

(14) $$d_{mes}^{\prime }=\frac{\sum_{i=1}^{b/2}\sum_{j=1}^{b/2}\left(\left\langle \mu_{i}^{c},\mu_{j}^{c}\rangle \;-\;\langle \mu_{i}^{c},\mu_{j}^{c^{\prime }}\right\rangle \right)\;+\;\sum_{i=b/2+1}^{b}\sum_{j=b/2+1}^{b}\left(\left\langle \mu_{i}^{c},\;\mu_{j}^{c}\rangle \;-\;\langle \mu_{i}^{c},\;\mu_{j}^{c^{\prime }}\right\rangle \right)}{\sqrt{2\sigma^{4}N\left[(b/2\;+\;2G{)}^{2}\;+\;\rho^{2}{\left(\frac{1-\rho^{b/2}}{1-\rho }\right)}^{4}\right]}},$$

where

$$G=\frac{(b/2\;-\;1)\rho \;-\;(b/2)\rho^{2}\;+\;\rho^{b/2+1}}{(1\;-\;\rho {)}^{2}}.$$

□

Compared to the microscopic scale, the structure of $d_{\text{mes}}^{\prime }$ reflects a number of key differences. The fact that time points are grouped into segments (of size b/2 in this case) and the corresponding temporal integration of raw neural activations introduce statistical interactions within each group. This changes the structure of the signal (numerator of Eq. (14)) from a linear sum to pairwise combinations within segments, leading to new contributions in the form of the inner products across time bins $\left\langle \mu_{i}^{c},\mu_{j}^{c}\right\rangle$. These cross-terms capture how similar signal components across nearby time bins contribute jointly, rather than independently. At the same time, noise (denominator of Eq. (14)) reflects correlated variability within each segment, as captured by the corresponding aggregation terms. Therefore, at both levels, the structure of the d-prime at the mesoscale replaces independent accumulation with localized (within-segment) integration.

Finally, the following result provides a closed-form expression for d-prime at the macroscopic extreme where neural activity is fully integrated into a single activation vector.

#### Theorem 3 (Sensitivity Index at the Macroscale).

Let $d_{\text{mac}}^{\prime }$ denote the sensitivity index defined in Eq. (4), with similarity statistics $P_{\text{mac}}^{c}$ and $P_{\text{mac}}^{x}$ defined in Eq. (3), trial hypervectors $v_{\text{mac}}^{c}$ defined in Eq. (2b), and neural responses following the statistical model in Eq. (5). Assume that Assumption 1 holds. Then,

(15) $$d_{mac}^{\prime }=\frac{\sum_{i=1}^{b}\sum_{j=1}^{b}\left(\left\langle \mu_{i}^{c},\;\mu_{j}^{c}\rangle \;-\;\langle \mu_{i}^{c},\;\mu_{j}^{c^{\prime }}\right\rangle \right)}{\sqrt{\sigma^{4}N{\left(b\;+\;2F\right)}^{2}}},$$

where

$$F=\frac{(b\;-\;1)\rho \;-\;b\rho^{2}\;+\;\rho^{b+1}}{{\left(1\;-\;\rho \right)}^{2}}.$$

At the macroscale, the aggregation is extended to the entire temporal window, so that all bins are combined into a single representation. Therefore, the signal term in the numerator of Eq. (15) now includes all pairwise interactions across time, accompanied by a respective change in the scaling of the noise term, ${\left(b+2F\right)}^{2}$, in the denominator. Notably, this squared form contrasts the linear form in the denominator of Eq. (8) and stems from the fact that temporal integration introduces interactions between all noise components and effectively couples variability across all times. Consequently, noise grows quadratically, making the macroscale highly sensitive to the structure of temporal correlations.

The above results establish how temporal aggregation reshapes both signal and noise contributions across scales. We next leverage these expressions to systematically compare sensitivity across resolutions and determine the parametric regimes in which aggregation enhances or limits discriminability.

### Comparison Across Temporal Scales

The analytical expressions provided in Theorems 1–3 allow us to analyze how d-prime varies across temporal scales for different values of signal and noise correlations. Before analyzing the general cases corresponding to arbitrary values of β and ρ, we begin with two limiting regimes that more clearly illustrate the contrasting roles of signal and noise correlations. Across all cases we assume the signal to be uncorrelated across conditions, namely, that $\langle \mu_{i}^{1},\mu_{j}^{2}\rangle =0$ for all i,j.

#### Case 1: uncorrelated signal and noise across time.

We begin with the extreme scenario where neither signal nor noise are correlated over time, namely,

β=ρ=0.

Substituting these into the expressions for the sensitivity index $d^{\prime }$ at the three scales (Eq. (8), Eq. (14), and Eq. (15)), yields

(16) $$d_{mac}^{\prime }=\frac{1}{\sqrt{2}}d_{mes}^{\prime }=\frac{1}{\sqrt{b}}d_{mic}^{\prime },$$

showing that both mesoscale and macroscale representations lead to a *reduction* in the sensitivity index relative to the microscale. In other words, in the absence of signal and noise correlations, averaging degrades decodability and raw neural activations at full resolution provide the highest amount of information about task conditions.

This observation can be explained based on how inner-product statistics are affected by temporal integration in the absence of correlations. At the microscale, the trial hypervector $v_{\text{mic}}^{c}$ preserves all constituent information, and the inner products in Eq. (3) for microscale, decompose into a sum of independent bin-wise contributions. Consequently, the difference between same-class and different-class inner products, i.e., $E\left(P_{\text{mic}}^{c}\right)-E\left(P_{\text{mic}}^{x}\right)$, accumulates linearly across bins, reflecting independent signal contributions at all time points. In contrast, at the meso and macro scales, temporal integration combines multiple bins into a reduced number of components. Given the lack of any signal correlations, this aggregation effectively cancels out independent contributions, reducing the total signal captured in the numerator of Eq. (4). On the other hand, the variance terms in the denominator of Eq. (4) scale linearly with b due to the additive accumulation of independent noises, leading to the deterioration of the signal-to-noise ratio encoded by d-prime under averaging.

#### Case 2: fully correlated signal but uncorrelated noise across time.

Next, consider the other extreme scenario where noise components still remain uncorrelated but signal remains perfectly correlated over time, namely,

ρ=0,β=1.

Note that when β=1, the two correlation structures in Eq. (7) coincide. It is straightforward to see that in this case signal must be constant, i.e.,

$$\mu_{i}^{c}\equiv \mu^{c},\;\;\text{for}\;\text{all}\;i,c,$$

Thus we obtain,

(17) $$d_{mac}^{\prime }=\sqrt{2}d_{mes}^{\prime }=\sqrt{b}\;d_{mic}^{\prime }.$$

These relations show that, in this case, temporal integration leads to an increase in the sensitivity index, with the macroscale achieving the largest gain. This behavior arises from how temporal integration interacts with the inner-product structure in the presence of aligned signal. In contrast to Case 1, integration at the meso and macro scales now combines bins that carry identical signal components. Therefore, signal contributions reinforce coherently across time, leading to a superlinear increase in the numerator of Eq. (4) through pairwise interactions between time points. At the same time, since noise remains uncorrelated across bins, the variance terms in the denominator of Eq. (4) still scale only through independent contributions. Consequently, noise grows more slowly compared to the coherently reinforced signal.

Together, Cases 1 and 2 represent two extreme scenarios with optimal condition decodability attained at, correspondingly, extreme scales. We next move to more general cases where signal and noise correlations can vary parametrically according to Eq. (6) and Eq. (7).

#### Case 3: persistent signal and decaying noise correlations over time.

We next consider the general regime in which noise correlations decay with an arbitrary rate ρ according to Eq. (6), while signal correlations remain persistent at an arbitrary value β following Eq. (7b). Assume, further, that the magnitude of signal is approximately constant, namely, that

(18) $$\left\|\mu_{i}^{c}\right\|\equiv \nu ,\;\;\text{for}\;\text{all}\;i,c.$$

Substituting these into the expressions for $d^{\prime }$ in Theorems 1–3 yields

(19a) $$d_{mic}^{\prime }=\frac{\nu^{2}b}{\sigma^{2}}\sqrt{\frac{1}{N(b\;+\;2E)}},$$

(19b) $$d_{mes}^{\prime }=\frac{\nu^{2}b\left(1\;+\;\left(\frac{b}{2}\;-\;1\right)\beta \right)}{\sigma^{2}\sqrt{2N\left[{\left(\frac{b}{2}\;+\;2G\right)}^{2}\;+\;\rho^{2}{\left(\frac{1-\rho^{b/2}}{1-\rho }\right)}^{4}\right]}},$$

(19c) $$d_{mac}^{\prime }=\frac{\nu^{2}b[1\;+\;(b\;-\;1)\beta ]}{\sigma^{2}(b\;+\;2F)\sqrt{N}}$$

where the variables E,F, and G are given in Theorems 1–3 and further depend on ρ. Unlike the previous two cases, the sensitivity index at neither scale is universally bigger than another. Instead, the relationship between each pair depends on the correlation parameters β and ρ. In particular, it can be shown that

(20a) $$d_{\text{mac}\;}^{\prime }>d_{\text{mic}\;}^{\prime }⟺{ℱ}_{b}^{\text{mac}>\text{mic}\;}(\rho ,\beta )>0,$$

(20b) $$d_{\text{mac}\;}^{\prime }>d_{\text{mes}\;}^{\prime }⟺{ℱ}_{b}^{\text{mac}>\text{mes}\;}(\rho ,\beta )>0,$$

(20c) $$d_{\text{mes}\;}^{\prime }>d_{\text{mic}\;}^{\prime }⟺{ℱ}_{b}^{\text{mes}>\text{mic}\;}(\rho ,\beta )>0,$$

where closed-form expressions for the functions $\left\{{ℱ}_{b}^{s,s^{\prime }}\right\}$ (which depend, notably, on the number of bins b) are provided in Supplementary Table S1. These inequalities provide a complete characterization of the parameter regimes governing the relative optimality of each scale.

**Figure 1.**
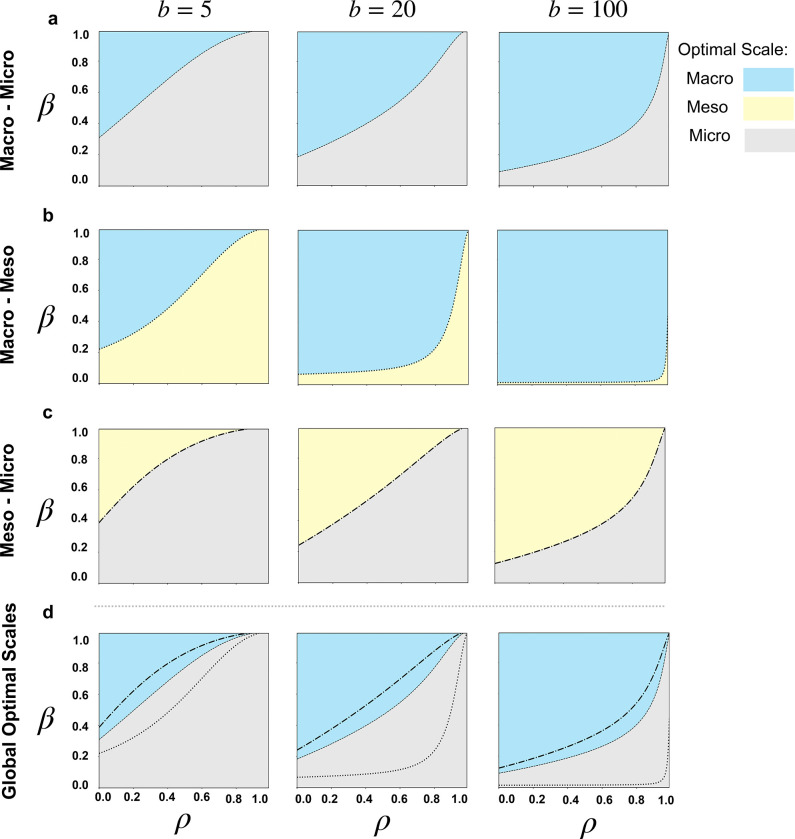
Optimal temporal resolution for information representation under decaying noise and persistent signal correlations. **(a)** Regional depiction of the pairwise comparison between micro and macro scales given by Eq. (20a) for varying numbers of bins b. The dotted curve indicates the correlation values where ${ℱ}_{b}^{\text{mac}>mic}=0$ and the colors denote the scale with greater sensitivity index (d-prime). **(b,c)** Similar to (a) but for the comparison between macro and meso scales given by Eq. (20b) and between meso and micro scales given by Eq. (20c), respectively. **(d)** Summary of panels (a-c) showing the overlaid comparison of all scales. While all three boundaries are shown for ease of comparison, colors denote the optimal scale at the respective (ρ,β) combinations.

Figure 1 demonstrates the precise boundary in the ρ−β plane where each comparison flips its direction, as well as a global illustration of which scale attains the greatest d-prime for each (ρ,β) combination. Notably, across all pairwise comparisons, the marked boundaries are monotonically increasing, giving rise to two regions in the ρ−β plane: a top-left region with relatively large β and small ρ, where more temporal integration is beneficial, and a bottom-right region with relatively small β and large ρ, where finer scales have greater d-prime. This behavior can be understood through the balance between signal and noise integration across temporal scales. When signal correlation (β) is large and noise correlation (ρ) is small, temporal integration largely preserves the shared signal but weakens (cancels-out) noise across time, resulting in improved signal to noise ratio and better decodability. In contrast, when noise correlation is larger than signal correlation, temporal integration has the opposite effect, making finer temporal scales more decodable.

In addition to the correlation parameters β and ρ, the relationship between scales depends notably on the number b of time points. In (Fig. 1a), as b increases (left to right). The boundary at which the inequality switches (i.e., ${ℱ}_{b}^{\text{mac}>\text{mic}}=0$) progressively shifts toward the bottom right, implying that temporal integration becomes more beneficial *for the same* (ρ,β) as the number of time points increases. This behavior occurs because the signal persists over time (cf. Eq. (7b)), allowing its effects to accumulate with integration over larger number of time points. Meanwhile, as the noise correlations decay over time, integration continues to have stronger weakening effects on noise over larger windows. A similar trend is observed across all pairwise comparisons (Figure 1b,c), reinforcing the presence of a strong and robust benefit in temporal integration over larger windows in the presence of persistent signal correlation.

When considering all three scales together, the optimal resolution falls—depending on the relative strength of signal and noise correlations—either at the micro or the macro scale, but never at the mesoscale (Figure 1d). This exclusive behavior shows the strength of the dichotomous tradeoff mentioned earlier, where the existence of a persistent signal correlation results in a marked difference between the effect of integration on signal and that on noise. As a result, the optimal $d^{\prime }$ is driven toward the two extremes, never favoring an intermediate resolution. This behavior is in contrast, in particular, to our empirical findings of mesoscale optimality in (Samiei et al., 2024), and motivated us to extend our framework to the more general case of Eq. (7a), as discussed next.

#### Case 4: decaying signal and noise correlations over time.

The preceding analysis illustrated the effect of temporal integration on neural representations within extreme regimes in which correlations and noise correlations were absent or remained constant across time. While these limiting cases and their universal optimality of extreme scales provide significant insight into the interplay between temporal integration and temporal signal/noise correlations, we next analyze the likely more realistic regime in which both signal and noise correlations decay with temporal distance following Eq. (6) and Eq. (7a). Substituting these into Eq. (8), Eq. (14), and Eq. (15), we obtain expressions analogous to Eq. (19), with the key difference that signal contributions now involve geometric sums of the form $\sum_{i,j}\beta^{\left|j-i\right|}$.

Interestingly, however, since the microscale sensitivity index in Eq. (8) does not involve average neural activity interaction terms across temporal bins, it remains unaffected by the introduction of such temporal correlations and $d_{\text{mic}}^{\prime }$ is still given by Eq. (19a). In contrast, both mesoscale and macroscale sensitivity indices are influenced by the presence of decaying temporal correlations in signal, and can be shown to be given by

(21b) $$d_{mes}^{\prime }=\frac{\nu^{2}\left[b\;+\;2\left(\frac{(b-2)\beta -b\beta^{2}+2\beta^{b/2+1}}{(1-\beta {)}^{2}}\right)\right]}{\sigma^{2}\sqrt{2N\left[{\left(\frac{b}{2}\;+\;2G\right)}^{2}\;+\;\rho {\left(\frac{1-\rho^{b/2}}{1-\rho }\right)}^{2}\right]}},$$

(21c) $$d_{mac}^{\prime }=\frac{\nu^{2}\left[b\;+\;2\left(\frac{(b-1)\beta -b\beta^{2}+\beta^{b+1}}{\left(1-\beta^{2}\right)}\right)\right]}{\sigma^{2}(b\;+\;2F)\sqrt{N}}.$$

where F and G are given in Theorems 2–3. Similar to Eq. (20), pairwise comparisons between these expressions can be simplified to

(22a) $$d_{\text{mes}}^{\prime }>d_{\text{mic}}^{\prime }\;⟺\;{𝒢}_{b}^{\text{mes}>\text{mic}}(\rho ,\beta )>0,$$

(22b) $$d_{\text{mac}}^{\prime }>d_{\text{mic}}^{\prime }\;⟺\;{𝒢}_{b}^{\text{mac}>\text{mic}}(\rho ,\beta )>0,$$

(22c) $$d_{\text{mac}}^{\prime }>d_{\text{mes}}^{\prime }\;⟺\;{𝒢}_{b}^{\text{mac}>\text{mes}}(\rho ,\beta )>0,$$

where the functions ${𝒢}_{b}^{s}$ are given in Supplementary Table S2.

**Figure 2.**
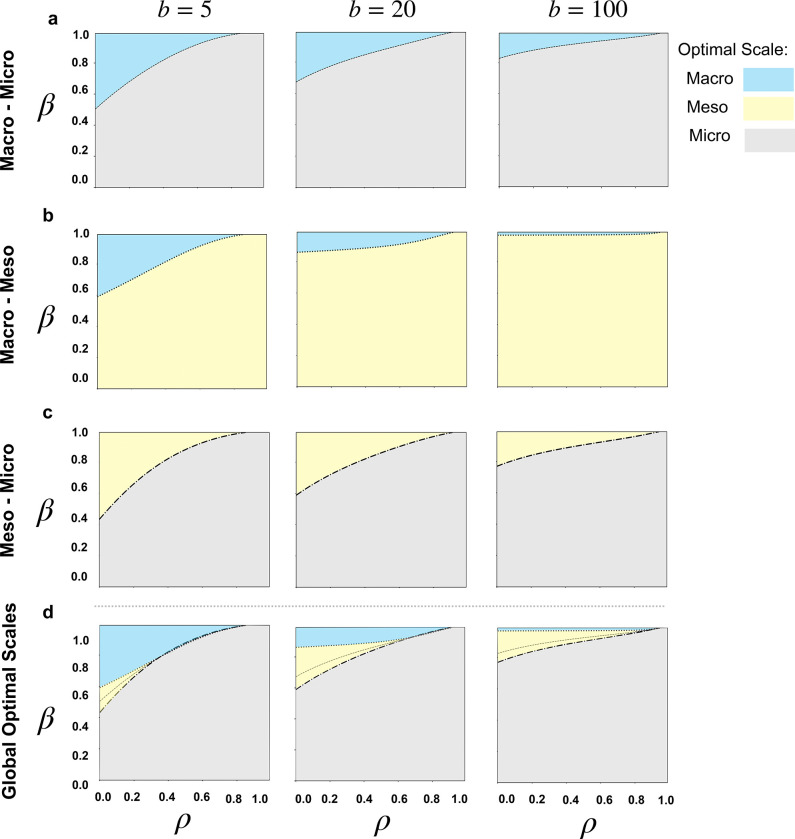
Optimal temporal resolution for information representation under decaying signal and noise correlations. **(a-c)** Regional depictions of the pairwise comparisons between micro, meso, and macro scales given by Eq. (22) for varying numbers of bins b. Details parallel those in Figure 1a–c. **(d)** Summary of panels (a-c) showing the overlaid comparison of all scales. Details parallel those in Figure 1d.

Figure 2a–c demonstrates the regions in the ρ−β plane where each inequality in Eq. (22) is (or is not) valid. Similar to Figure 1, all three pairwise boundaries (i.e ${𝒢}_{b}^{s}=0$) remain monotonically increasing, dividing the parameter space into high ρ/lowβ and low ρ/highβ regions where the finer and coarser scales achieve greater decodability, respectively. In contrast to Figure 1, however, increasing the number of time points b now has the effect of shifting all pairwise boundaries upwards, making d-prime greater at finer scales. This indicates that temporal integration is more likely to become 'excessive' when signal correlations decay over time, an effect that is consistent with the larger signal cancellation that takes place as a result of temporal averaging under decaying correlations.

Most notably, unlike the case with persistent signal correlations, the mesoscale now emerges as the optimal resolution in the summarized comparison across all three scales (Figure 2d). While the microscale dominates in most parameter regimes and the macroscale optimality region shrinks with increasing b, the mesoscale optimality region persists across all b. The latter further grows with increasing b, particularly in biologically realistic parameter regimes characterized by slow decay in signal correlations and fast decay in noise correlations. This finding is remarkable given our recent observation of the optimality of the mesoscale in real neural data (Samiei et al., 2024). Overall, our results demonstrate that introducing decaying signal correlations fundamentally reshapes the optimality landscape, giving rise to a robust intermediate regime under biologically realistic signal-to-noise conditions, whereby mesoscopic averaging provides the optimal balance between capturing coherent information-bearing signal while averaging over task-irrelevant fluctuations.

## DISCUSSION

### Summary.

In this work we developed a rigorous theoretical framework showing that the optimal temporal scale of information representation in neural populations is determined by the balance between microscopic signal correlations (MSC), microscopic noise correlations (MNC), and the way both decay across time. Through an explicit analysis of the decodability of task conditions from temporally integrated population activity at different scales, we showed that different correlation regimes give rise to different optimal scales of representation. Most notably, in the extreme cases where signal and noise correlations are either absent or persistent throughout the trial duration (Cases 1–3, see Results), the optimal resolution falls at one of the two extremes: macroscale (microscale) if signal correlations are significantly larger (smaller) than noise correlations. On the other hand, if both signal and noise correlations decay with time (Case 4), mesoscale representations also become optimal for a robust and realistic range of parameters: where signal correlations decay slowly while noise correlations decay significantly faster.

Our results clearly demonstrate a fundamental phenomenon where temporal integration is a double-edged operation for neural representations: it can improve task-relevant information representation by averaging out noise across time, but it can also weaken such information by canceling signal components that are only weakly correlated across temporal bins. This effect can be seen most strongly when both signal and noise correlations decay with temporal separation (Case 4, see Results). In this case temporal integration gives rise to a genuine trade-off: modest integration enhances signal-to-noise ratio and decodability, whereas excessive integration disrupts representational structure and lowers decodability. In this biologically more realistic regime, mesoscale representations emerge as optimal.

These results further provide a theoretical explanation for our recent empirical observations of mesoscale optimality in neural systems (Samiei et al., 2024). Rather than being an intrinsic property of neural coding, mesoscale optimality arises as a consequence of structured temporal correlations. More broadly, our results highlight that temporal averaging is not merely a preprocessing step, but a fundamental operation that reshapes neural information in a structured and predictable manner.

### Role of signal–noise interactions.

Our main analytical results are derived under the simplifying Assumption 1 that signal and noise components are uncorrelated. This assumption allows the sensitivity index to decompose explicitly into signal and noise contributions and therefore leads to tractable closed-form expressions. Under this setting, the sensitivity index effectively behaves as a signal-to-noise ratio, where signal accumulation and noise variance (numerator and denominator of d-prime, respectively) can be analyzed independently across temporal scales.

Nevertheless, our general approach and theoretical framework can be extended to the general setting where signal and noise components are not necessarily independent. An example of such extension is provided in Supplementary Note 2 for the case of two time points (b=2). Interestingly, while relaxing Assumption 1 introduces signal–noise interaction terms which make closed-form expressions considerably more cumbersome, it does not qualitatively change the model's behavior. Within this setting, the sensitivity index depends explicitly on both signal magnitude ($\nu^{2}$) and noise magnitude ($\sigma^{2}$). When noise dominates ($\sigma^{2}\gg \nu^{2}$), the behavior closely matches the results obtained under Assumption 1. In contrast, when signal dominates ($\nu^{2}\gg \sigma^{2}$), temporal averaging becomes uniformly beneficial. For intermediate cases (e.g., ν=σ=1, Supplementary Figure S1a), the general model shows a stronger preference for averaging compared to the simplified case, reflecting the contribution of signal–noise interaction terms. Although the optimality curves for averaging and non-averaging still intersect, the resulting feasible regions are slightly shifted relative to the setting with no signal-noise interactions (Supplementary Figure S1).

### Implications for neural data analysis.

Our results have direct implications for how neural data should be analyzed and interpreted. Many standard preprocessing techniques—such as temporal binning, smoothing, and dimensionality reduction—implicitly perform temporal averaging. Our framework shows that the effectiveness of such operations in ‘cleaning the signal’ and extracting meaningful task-relevant dynamics from task-irrelevant noise depends critically on the temporal correlation structure of the data. In particular, in task conditions and brain structures where signal correlations persist much longer than noise correlations (e.g., under stationary tasks or within higher-order association cortices), averaging improves signal to noise ratio up to significantly coarser scales compared to task conditions and brain structures where signal correlations decay relatively fast (e.g., under highly dynamic tasks or within early sensory structures). More broadly, our result suggests that optimal preprocessing pipelines should not rely on fixed temporal scales, but should instead adapt to the underlying signal and noise statistics of the data, and the theoretical conditions derived in this work provide a principled basis for such adaptations.

### Limitations and future directions.

For analytical tractability, we considered a restricted set of assumptions. First, we assumed signal and noise correlations that were either zero, constant, or exponentially decaying. While these capture key regimes of temporal dependence, real neural activity may exhibit more complex correlation structures. Second, we assumed noise to be independent across neurons and focused solely on statistical dependence over time. Extending the framework to incorporate spatial correlations and network structure would provide a more complete picture of multiscale neural representations and remains an important direction for future work.

A further natural extension of this framework is to examine whether and to what extent the predicted optimal scales vary across recording modalities. In our earlier empirical work using single-unit spiking activity recorded via NeuroPixel probes Samiei et al. (2024), we observed specific mesoscopic spatial and temporal scales to be optimal for decoding visual stimulus identity. The present theory suggests that those optimal resolutions are not universal, but instead depends on the underlying structure of signal and noise correlations that may differ across data modalities. Testing this prediction (both directly and via a combination of results developed here and quantification of correlation decay rates) in diverse data modalities can shed light on interesting interplays between optimal spatiotemporal resolutions, data modality, and temporal dynamics.

## METHODS

Detailed proofs of our analytical results (Theorems 1–3), constituting the core part of our methodology, are provided in Supplementary Note 1. In this section we provided additional details about the computational methods we used to interpret the results of these Theorems and compare decodability across scales.

### Analytical derivation of pairwise comparison functions across scales.

To characterize the relative optimality of different temporal scales, we formulated pairwise comparisons in terms of inequality conditions between the corresponding sensitivity indices. Specifically, we defined optimality condition functions in Eq. (20) and Eq. (22), with closed-form expressions given in Supplementary Tables S1 and S2, respectively.

These functions are obtained by substituting the closed-form expressions of $d^{\prime }$ into the pairwise differences and simplifying the resulting expressions. To ensure the validity of these inequalities, we analyzed the dominant terms of each expression and examined their dependence on the parameters ρ and β. In particular, we verified that the leading-order contributions remain positive across the parameter regimes considered. Formally, we computed the following partial derivatives and verified that they satisfy

$$\frac{d}{d\beta }({ℱ}_{b}^{s>s^{\prime }})>0\;\;\text{and}\;\;\frac{d}{d\beta }({𝒢}_{b}^{s>s^{\prime }})>0\;\;\text{for}\;\text{all}\;\;s,s^{\prime },b\geq 2.$$

This confirms the monotonic behavior of the expressions and therefore ensures that the sign of each inequality is preserved within the relevant domain.

### Numerical evaluation and visualization of optimality regions.

To visualize the analytically derived optimality conditions, we evaluate the functions ${ℱ}_{b}^{s>s^{\prime }}(\rho ,\beta )$ and ${𝒢}_{b}^{s>s^{\prime }}(\rho ,\beta )$ (cf. Supplementary Tables S1 and S2) numerically over a uniform grid defined by ρ∈[0,0.99],β∈[0,0.99], and a discretization of 300 × 300 points (no numerical root-finding). For each grid point, the sign of the corresponding function determines the optimal regime, with the convention that

$${ℱ}_{b}^{s>s^{\prime }}(\rho ,\beta )>0\Rightarrow d_{s}^{\prime }>d_{s^{\prime }}^{\prime },$$

and vice versa. The resulting regions were then visualized using filled contour plots, where Boolean masks identify parameter regions corresponding to each optimal regime. The boundaries between regimes are given by the zero level sets

$${ℱ}_{b}^{s>s^{\prime }}(\rho ,\beta )=0,$$

which were approximated numerically as contour lines on the discretized grid. To avoid numerical instabilities associated with singularities in the analytical expressions, the parameter range is restricted to ρ<1. The same process was repeated for the comparison functions ${𝒢}_{b}^{s>s^{\prime }}$ visualized in Figure 2.

## Supplementary Material

Supplement 1
